# Inflammatory Bowel Disease

**DOI:** 10.1155/2012/341052

**Published:** 2012-03-27

**Authors:** David B. Sachar, Derek Jewell, Christoph Gasche, Jonathan Braun

**Affiliations:** ^1^Mount Sinai School of Medicine, New York, NY 10029, USA; ^2^Nuffield Department of Clinical Medicine, John Radcliffe Hospital, Oxford OX3 9DU, UK; ^3^Medical University of Vienna, A-1090 Vienna, Austria; ^4^Department of Pathology and Laboratory Medicine, David Geffen School of Medicine at UCLA, Center for Health Sciences, 13-222 Los Angeles, CA 90095, USA

Studies of inflammatory bowel disease (IBD) over many decades have spanned a wide range of topics from clinical epidemiology and diagnosis, through dietary and pharmacologic therapy and immunologic and biologic etiology, to pathophysiologic links to malignancy. In this special issue devoted to IBD, the Guest Editors and one of the authors have selected a series of seven papers from seven countries that reflect this entire spectrum.

Leading off with clinical epidemiology, Y. Correa et al. from the University of Puerto Rico call our attention to the increasing prevalence of both ulcerative colitis (UC) and Crohn's disease (CD) among Hispanics. Particularly noteworthy is their observation that no particular phenotypic features consistently distinguish this Puerto Rican cohort from other white, non-Hispanic populations. Could we take these findings to infer that environmental factors are playing a role in the presentation of IBD that trumps or at least balances the role of genetics?

From clinical epidemiology in Puerto Rico, we move to clinical diagnosis in Italy. E. Calabrese et al. at the University of Rome offer us a review of the utility of contrast-enhanced ultrasound of the small bowel in the evaluation of patients with CD. With or without radiation, both CT and magnetic resonance (MR) enterography are relatively expensive and labor-intensive, so a lower-cost, more convenient alternative could be a welcome addition to the armamentarium. Will contrast-enhanced ultrasound fill this role?

No survey of IBD could be complete without at least some attention to therapy. G. M. Fung and A. Szilagyi from McGill University in Montreal caution us against too readily embracing the fad of carbohydrate withdrawal as dietary treatment for the symptoms of IBD. They remind us of the potential importance of carbohydrates as a substrate for bacterial production of short-chain fatty acids, which are possibly critical in maintaining anti-inflammatory homeostasis in the intestinal tract.

Another paper in this special issue shifts from dietary to pharmacologic therapy. While anti-TNF and anti-integrin molecules have been occupying most of the spotlight for IBD treatment in the past decade, novel approaches targeting other pathways will undoubtedly yet emerge. L. R. Fitzpatrick from Penn State College of Medicine calls attention to one of the key alternative pathways in his review of the IL-23/IL-17 axis. Certainly, it has been a seminal discovery that these two closely intertwined pathways can both be neutralized by targeting the common p40 subunit of IL-12 and IL-23. L. R. Fitzpatrick's review shows us that several different approaches to targeting this central inflammatory pathway, already usefully exploited in treating psoriasis, might well prove beneficial in IBD as well.

The paper by T. L. Holm et al. from Denmark moves from the bedside to the bench by employing a murine model of CD. Using the severe combined immunodeficiency disease (SCID) adoptive T-cell transfer model of colitis, these authors provide an intriguing link to L. R. Fitzpatrick's review. It turns out that, among an array of eight different agents tested for prevention or treatment of this form of experimental colitis, only anti-IL-12p40 and abatacept (cytotoxic T-lymphocyte antigen 4 immunoglobulin) induced remission of established disease. To be sure, this murine system is far from a perfect model of human IBD, but the Danish study is certainly a confirmation of the theoretical anti-inflammatory potential of targeting the IL-12/23 pathway.

The penultimate paper concentrates on the newest candidate etiology of IBD, namely, intestinal dysbiosis. G. De Hertog et al. from Leuven, Belgium, used laser microdissection of intestinal tissues from four CD patients, six inflammatory disease controls, and three noninflamed controls to demonstrate “significant changes of the composition, abundance and location of the gut microbiome in [Crohn's] disease.” This avenue of research is clearly going to be a major focus over at least the next decade. 

Finally, our special issue presents a review of several inflammation-associated colorectal cancer model developed by T. Tanaka from Japan. He expresses the hope “that use of these models will advance elucidation of the mechanisms (methylation and microRNA) of inflammation-associated colorectal carcinogenesis, exploration of its suppression and mechanisms, and clarification of the mechanisms of tumor-promotion activity of DSS [dextran sodium sulfate].”

We readily acknowledge that the seven articles selected by the three indefatigable Guest Editors and me from the many manuscripts submitted will not provide definitive answers to the mysteries of IBD. Nonetheless, this special issue makes a creditable effort to identify some of the most important questions.

*David B. Sachar* *David B. Sachar* *Derek Jewell* *Derek Jewell* *Christoph Gasche* *Christoph Gasche* *Jonathan Braun* *Jonathan Braun*

